# Central effects of acetylsalicylic acid on trigeminal-nociceptive stimuli

**DOI:** 10.1186/1129-2377-15-59

**Published:** 2014-09-09

**Authors:** Inga L Kröger, Arne May

**Affiliations:** 1 Department of Systems Neuroscience, University Medical Center Hamburg- Eppendorf, Martinistr. 52, Hamburg D-20246, Germany

**Keywords:** Aspirin, ASA, Trigeminal-nociception, fMRI, Pain, Pharmacological modulation, Cingulate cortex

## Abstract

**Background:**

Acetylsalicylic acid is one of the most used analgesics to treat an acute migraine attack. Next to the inhibitory effects on peripheral prostaglandin synthesis, central mechanisms of action have also been discussed.

**Methods:**

Using a standardized model for trigeminal-nociceptive stimulation during fMRI scanning, we investigated the effect of acetylsalicylic acid on acute pain compared to saline in 22 healthy volunteers in a double-blind within-subject design. Painful stimulation was applied using gaseous ammonia and presented in a pseudo-randomized order with several control stimuli. All participants were instructed to rate the intensity and unpleasantness of every stimulus on a VAS scale. Based on previous results, we hypothesized to find an effect of ASA on central pain processing structures like the ACC, SI and SII as well as the trigeminal nuclei and the hypothalamus.

**Results:**

Even though we did not find any differences in pain ratings between saline and ASA, we observed decreased BOLD signal changes in response to trigemino-nociceptive stimulation in the ACC and SII after administration of ASA compared to saline. This finding is in line with earlier imaging results investigating the effect of ASA on acute pain. Contrary to earlier findings from animal studies, we could not find an effect of ASA on the trigeminal nuclei in the brainstem or within the hypothalamic area.

**Conclusion:**

Taken together our study replicates earlier findings of an attenuating effect of ASA on pain processing structures, which adds further evidence to a possibly central mechanism of action of ASA.

## Background

Acetylsalicylic acid (ASA), corresponding to the group of non-steroidal anti-inflammatory drugs (NSAIDs), is until today one of the most frequently used analgesics for pain syndromes and widely applied in the treatment of headache. The efficiency of 500-1000 mg ASA administration in the treatment of acute migraine attack has been proven in several clinical trials [1-3] and there are also studies showing a prophylactic effect of ASA on migraine without aura [4]. In 1971 its inhibitory effect on local prostaglandine synthesis was discovered [5,6]. This mechanism of action is mainly driven by its inhibitory effect on the enzyme cyclooxygenase which is subdivided into two isoenzymes cyclooxygenase-1 (COX-1) and cyclooxygenase-2 (COX-2) [7-10]. Because traditional NSAIDs like Acetylsalicylic acid inhibit both COX-1 and COX-2 isoenzymes, which may lead to adverse gastric side effects, more specific COX-2 inhibitors have been introduced [11,12]. Both isoenzymes are expressed in peripheral tissues and the central nervous system (CNS) (for review see [7]) and although a solely peripheral mechanism of action was suggested for several years, various studies suggested an additional central effect of ASA [13-20]. Hence, next to the inhibition of peripheral prostaglandine synthesis, a central mechanism, respectively the interaction of both, is the most likely mode of action [15,21]. In the acute migraine attack ASA could also be shown to attenuate accompanying symptoms like nausea or photophobia [22,23], again suggesting central nervous effects. In addition ASA exhibits an inhibitory effect on the activation of central trigeminal neurons after sagittal sinus stimulation [19,24]. Moreover, a high binding affinity of ASA to the dorsal horn and nuclei in the brainstem could be observed [19]. Conversely, a very early study by Shyu & Lin explored a possible effect of ASA on painful tooth stimulation in conscious monkeys, finding a significantly elevated pain threshold after ASA administration into the anterior hypothalamic area but not after injection of ASA into the periaqueductual grey matter or dorsal raphe region [14]. As this effect could be blocked by prestimulus blockade of serotonin receptors within the hypothalamic regions, the authors suggested a central serotonergic mechanism of action for ASA within this area [14]. The involvement of serotonin receptors in the analgesic effect of ASA has also been proposed by other studies [25].

Recently, using fMRI the neuronal correlates of analgesic (acute pain) and antihyperalgesic (mechanical hyperalgesia) conditions were investigated and pharmacologically modulated using a nonselective COX-inhibitor (ASA) and a selective COX-2 inhibitor (parecoxib) [21,26]. Following ASA, a decreased activation of the primary (SI) and secondary somatosensory cortex (SII) [21] and a slight decrease in the anterior cingulate cortex [26] could be revealed in the acute pain model. These effects were stronger in the hyperalgesic model, where a stronger activation for placebo compared to ASA was found in several brain areas including SI, bilateral SII, insula, middle and inferior frontal cortices as well as the parietal association cortex [21].

Based on these results we investigated the influence of 500 mg ASA (i.v.) on trigemino-nociceptive stimulation using a previously described standardized design for functional magnetic resonance imaging (fMRI) [27]. As trigeminal-nociceptive stimulation causes acute pain, we mainly hypothesized to find an effect of ASA on SI, SII or the anterior cingulate cortex as reported earlier [21,26] and additionally investigated a possible effect of ASA on neuronal activation in the hypothalamus and the trigeminal nuclei [14,24].

## Methods

### Subjects

We used fMRI to investigate 22 healthy subjects in a randomized, double-blind, placebo-controlled, cross-over study. All participants were devoid of migraine history or any other neurological, internal or psychiatric disorder. Participants were also controlled for having any history of pain syndrome, as well as acute minor pain (e.g. tooth-ache) and possible contraindication for ASA. None of the volunteers took any type of medication. Participants not fitting these criteria were not included. Prior to fMRI data acquisition, all volunteers were informed in detail about the purpose of the study and the possibility to withdrawal from the experiment at any time. Remuneration was paid for study participation. The study was approved by the local Ethics Committee and conducted in accordance to the declaration of Helsinki.

### Study design

All participants underwent two separate fMRI sessions whereof the second fMRI scan was conducted at least 8-14 days after the first session to control for wash-out effects. Prior to the first fMRI scan, all participants underwent a training session outside the scanner to get used to the study paradigm which is a slightly extended version of a successfully used experiment described in detail elsewhere [27]. In a nutshell, during the fMRI scan all volunteers received 15 standardized trigemino-nociceptive stimuli (gaseous ammonia) which were applied through a thin Teflon tube (connected to the olfactometer outside of the scanner) into the left nostril of the participants. Furthermore, volunteers perceived several control stimuli during the event- related fMRI consisting of olfactory (rose odor) as well as an odorless (air puffs) stimulation and a visual stimulus (flickering checkerboard). The latter being, in contrast to the intranasal applied stimuli, presented on the screen in front of the participants for 4 seconds. Presentation of the stimuli took place in a pseudo-randomized order, in order to control for trigeminal stimuli not to be followed by each other. Subsequently, all subjects had to rate the intensity (from 0 (no pain/no sensation to 100 (highest pain imaginable/highest intensity imaginable)) and pleasantness (from -50 very pleasant to 50 very unpleasant) of the stimulus on a visual analog scale (VAS). Preceding the next run, an inter-trial interval (ITI) of 4-6 seconds followed. All volunteers were instructed to breath orally throughout the entire experiment.

### Medication (ASA)

The intravenous administration of 500 mg ASA or saline (0.9%) was assigned in a randomized order between participants. Thus, half of the subjects received saline during the first fMRI data acquisition whereas the second half of the volunteers received the medication during the first fMRI data acquisition. Consequential, after a wash-out phase of 8-14 days the correspondent substance (verum or saline) was administered before the second fMRI session. The study was conducted under double-blind conditions, i.e. the administration of the drug was randomized and done by an independent doctor. ASA was administered approx. 15 minutes (14-17 minutes) prior to scanning. This time window was chosen because it could be shown earlier that while the first exponent of ASA has a half-life of 2.8 minutes, the second exponent of ASA has a half-life of 15 minutes [28]. Because the cyclooxygenase inhibition of ASA is irreversible, C_max_ of ASA does not play such a crucial role for our study and since SA has a very long half life time of up to 4 hours, we can be sure that the effect lasts for the duration of our study.

### Image acquisition

Anatomical and functional data were collected using a 3 T Scanner (Siemens-Trio) with a 32 channel head coil. The protocol included a high resolution T1-weighted structural image (voxel size 1×1×1 mm) for every participant using a Magnetization Prepared Rapid Gradient Echo (MPRAGE) sequence as well as T2*-weighted images using an echo-planar imaging sequence in an axial order (Time of Repetition: 2.62 s, echo time: 30 ms, flip angle 80°, field of view: 220×220 mm, 40 axial slices, slice thickness 2 mm, gap of 1 mm). Every session consisted of 855-1202 volumes (mean 965.7 volumes). Differences in volumes acquired were due to time differences within the rating procedure of the subjects. The fMRI protocol was an event-related design. Because of our special interest in the brainstem we chose the caudal part of the cerebellum in each subject for use as a point of reference to the lowest slice which also refers to an earlier study [27].

### Behavioral data analysis

All analyses regarding behavioral data were performed using SPSS (Statistical Program for Social Sciences; Version 22.0). We calculated averaged pain ratings subsequent to trigemino-nociceptive stimulation for every subject in every session. A paired t-tests was performed to compare pain intensity ratings between sessions (medication vs. saline) using a statistical threshold of p < 0.05.

### Statistical analysis for imaging data

Imaging data was processed and analyzed using statistical parametric mapping (SPM8; Wellcome Department for Imaging Neuroscience, London, UK). Individual functional scans were preprocessed including the following steps: slice time correction, data realignment of all volumes to the first volume, co-registration and normalization into the MNI (Montreal Neurological Institute) stereotactical space as well as smoothing using an isotropic 8 mm full-width at half-maximum (FWHM) three- dimensional Gaussian kernel. Thereafter, statistical analyses on a single subject level were performed using a general linear model (GLM). The different events “Ammonia” (trigemino-nociceptive stimulation), “rose odor”, “air puffs”, “visual stimulation” and “button presses” were therefore modeled as delta functions convolved with the default SPM8 canonical hemodynamic response function. Additionally six movement parameters (translation and rotation around x-, y-, and z-axis) were included in the 1^st^-level design matrix of every subject as regressors of no interest. As the major interest of our study concerned the different effect of medication or saline on painful stimulation, contrast images for every subject were generated to identify regions showing increased activation during painful stimulation compared to air puffs in medication vs. saline conditions [Saline session (“ammonia > air puffs”) > Medication session (“ammonia > air puffs”)]. A t-statistic was calculated for every voxel and contrast images were inserted into a random effects second level model (one-sample t-test) for group analyses. This was conducted to assess BOLD signal differences in response to painful stimulation between medication and saline condition. Assuming that ASA could have a central effect on pain related structures as the anterior cingulate cortex, SI or SII as indicated by earlier studies [21,26], we were especially interested in the responses of these regions to trigeminal-nociceptive stimulation. Therefore, we conducted small volume correction using family wise error correction (FWE p<0.05) for the anterior cingulate cortex, SI and SII. Specifically, we set the threshold to p <0.05 FWE corrected for the anterior cingulate using a mask of the right and left anterior cingulate cortex from the Harvard-Oxford cortical/subcortical structural atlas (http://www.cma.mgh.harvard.edu/fsl_atlas.html). For regions of interest that are not explicitly defined in the Harvard-Oxford cortical/subcortical structural atlas (SI, SII) small volume corrections (FWE-corrected, *p* < 0.05) were performed using 16 mm spheres centered on coordinates [x = ±52, y = -26, z = 18] for SII and [x = ± 36, y = -30, z = 54] for SI taken from prior research [21,26] after converting them from Talairach in MNI space . As previous animal studies found an effect of ASA on the anterior hypothalamic area and the trigeminal nuclei, we again performed small volume corrections (FWE-corrected, *p* < 0.05) using a 6 mm radius sphere for the trigeminal nuclei centered on coordinates [x = 6, y = -39, z = -45; x = -9, y = -39, z = -45] of a previous study [29] adjusted to the setting of our scanner ( left : x = -10, y = -40, z = -46; right: x = 6, y = -40, z = -46). For the hypothalamus we also used a sphere of 12 mm radius centered on coordinates [x = 0, y = -2, z = -8] as reported in the Talairach atlas and used in previous studies [30] after converting them in MNI space.

## Results

Two subjects had to be excluded from data analysis because they did not show a clear painful response to trigemino-nociceptive stimulation (mean pain rating in both sessions <35). One participant withdrew from the study. Therefore, our data analysis (behavioral as well as fMRI data) is restricted to 19 healthy subjects.

### Behavioral data

Mean Pain intensity rating (±SEM) for the ASA session was 61.7 ± 2.73 and for the saline session 62.1 ± 2.71. A paired t-test comparing the average pain ratings of both sessions failed to reach significance (p > 0.05).

### Imaging data

#### *Main effects of painful trigeminal stimulation*

Pooling both sessions together (medication and saline session) we replicated earlier findings (of studies using the same paradigm) [27,29,31] of a statistically significant increase in neuronal activation in several pain related cortical and subcortical brain areas following trigemino-nociceptive stimulation. This statistical significant increase (FWE corrected) in BOLD signal changes included the middle and anterior cingulate cortex, the insular cortex, thalamus, the cerebellum as well as somatosensory cortices.

### Effect of ASA on trigeminal stimulation compared to Saline

**Figure 1:**
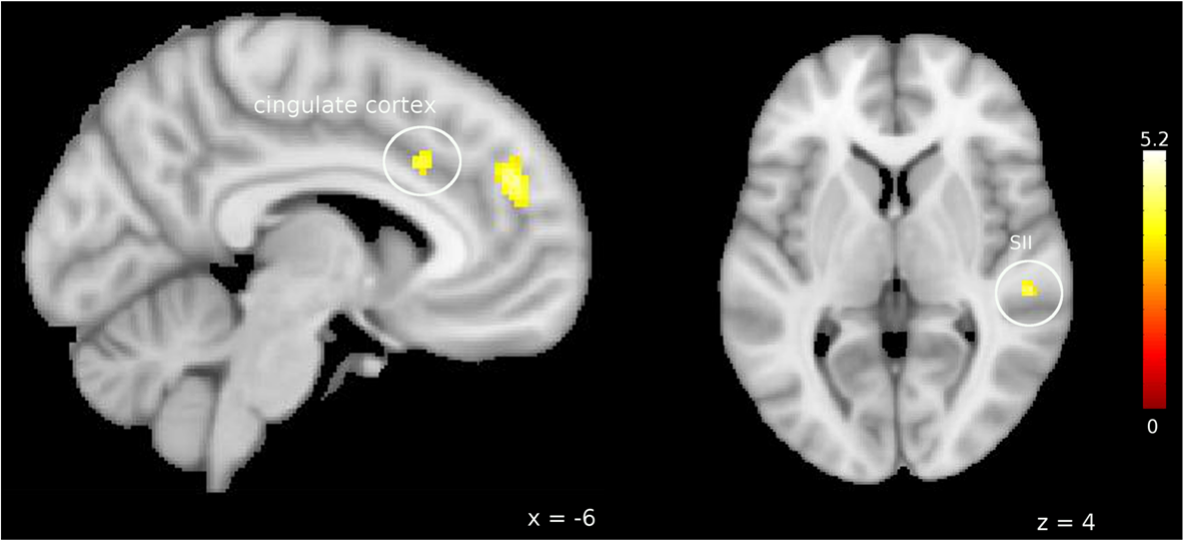
**BOLD signal intensity during painful stimulation: Saline > ASA.** Increased BOLD signal intensity in the anterior cingulate cortex (left) and secondary somatosensory cortex (right) during nociceptive input (ammonia > air puffs) after saline treatment compared to ASA condition. Visualization threshold is set to p < 0.001 (uncorrected). Bold activation is projected onto a MNI152_T1 brain template (FSL). Color scales show T values of individual voxels.

As shown in Figure 1, we found an increased activation in the left ACC [x = -6, y = 20, z = 32; T_(18)_ = 4.13, p = 0.029 (FWE corrected)) as well as in the right SII [x = 54, y = -28, z = 4, T_(18)_ = 4.4 p = 0.047 (FWE corrected)] regarding the contrast [Placebo (Ammonia > air puffs) > ASS (Ammonia > air puffs)] replicating the trend found in a previous study [26]. Contrary to earlier results, we did not reveal any significant activation in SI (p > 0.05 (FWE corrected)). Additionally, no increased activation for placebo compared to ASA condition could be revealed regarding the hypothalamic area or the trigeminal nuclei. No significant differences could be revealed for the opposite contrast (ASA > Saline).

## Discussion

Finding a significantly decreased activation of ACC and SII in response to trigeminal nociceptive stimulation in the ASA condition compared to placebo (Figure 1), we could replicate a trend shown in previous studies indicating a possible central mechanism of ASA not only on mechanically induced hyperalgesia but also on acute pain [21,26]. The ACC as well as SII are structures frequently found during nociceptive stimulation and known to play a crucial role in the processing of pain [32-36]. While the ACC has been attributed among others to reflect an emotional pain component [37,38], it is also crucially involved in antinociception and anticipatory anxiety [39]. SII, also shown to be modulated by ASA, is related to pain intensity coding [36] and seems to be responsible for the integration of salient (painful and nonpainful) somatosensory stimuli [40]. However, we did not observe any behavioral effect of ASA on pain intensity perception of the volunteer. This lack of a behavioral effect combined with an obtained CNS effect of ASA on painful stimulation is in line with an earlier study using mechanical pain [21] and painful carbon dioxide pulses delivered to the nasal muscosa [17]. More evidence for a central effect of ASA on pain processing is coming from an imaging study investigating pain ratings as well as stimulus evoked responses to nociceptive stimulation using an electroencephalogram (EEG). In this case, oral administration of 1000 mg ASA reduced the overall pain ratings as well as all pain related cerebral potentials significantly [13]. Of note, effects of ASA increased with time, meaning that ASA showed an attenuating effect compared to placebo on all relevant variables during the experiment. But this effect did not reach significance before 90 minutes post medication [13]. Additionally, a study using electrical brain potentials could show an effect of ASA on late but not early waveforms of the evoked potentials in healthy man, which are suggested to reflect pain perception processing [18]. Nonetheless, investigating a possible difference between later and earlier VAS scores of ammonia, we could not find a significant difference within the ASA condition. However, as these earlier studies did not specifically investigate the effect of ASA on migraine, the exact mechanism of action how ASA reduces head pain is still controversial. Knowing that the trigeminal innervation plays a pivotal role in migraine, several studies investigated the effect of ASA on trigeminovascular nociceptive input [19,24]. Especially the use of an electrophysiological animal model of superior sagittal sinus stimulation [24] was promising to gain further insight in the mechanisms of actions through which ASA exerts its clinical effect in migraine, as it reflects migraine-like pain or neuropeptide release similar to that in an acute attack [41]. An inhibitory effect on the activation of central brainstem nuclei [19] as well as reduced peak-to-peak amplitudes for trigeminal somatosensory evoked potentials in the dorsolateral spinal cord [24] could be observed after ASA administration. Interestingly, naloxone did not invert the inhibitory effect on the brain evoked potentials [24]. Furthermore, this effect was not due to a peripheral blockade of inflammatory induced neuropeptides [24], proposing a central inhibitory mechanism of action for ASA on the trigeminovascular system [19,24]. Nonetheless, in the present study we could not reveal a significant difference in BOLD signal changes in response to trigeminal-nociceptive stimulation in the trigeminal nuclei after ASA administration compared to the saline condition. We note, that ASA and triptans inhibit the nociceptive blink reflex in the acute migraine attack, but seem to have no effect on trigeminal pain in migraineurs in the interictal state or in healthy volunteers [42], suggesting a modulatory effect on the trigeminal nociceptive system which occurs only in the migraine attack but not in the healthy system nor in the interictal phase [42], for example by blocking sensitization.

In 1988 it was shown that administration of acetylsalicylate of lysine leads to an increase in 5-hydroxyindoleacetic acid in the hypothalamus and the brainstem [43]. Investigating the effect of aspirin on the inhibitory antinociceptive brainstem reflex, another study obtained a significant effect of ASA especially on the latency of the early suppression period (ES1) within this exteroceptive suppression of electrical activity in the temporal muscle [44]. While ES1 latency was decreased in patients suffering from a primary headache disease after administration of ASA, it was increased in healthy participants [44]. Moreover, ASA caused a significant growth in ES2 (late suppression period) duration [44], whereas placebo failed to show this increase. An effect of ASA on complex pain control mechanisms was suggested [44]. We did not find any significant difference in BOLD signal changes in response to trigeminal-nociceptive stimulation in the hypothalamus of healthy volunteers after ASA administration compared to saline condition. However, the dosage of ASA used in the present study differs from earlier used dosages [21]. Even though we chose to administer 500 mg ASA (i.v.) because there is strong evidence showing its clinical effectiveness in the acute migraine attack [1], the divergence between dosages could at least partly explain the obtained differences in neuronal activation. Finally, ASA reveals its effect by inhibiting COX-1 and COX-2 enzymes that lead to the expression of prostaglandins which play a crucial role within the inflammatory process [7,8]. It is therefore to be expected that the effect of ASA is much more pronounced in the state of hyperalgesia compared to acute pain [21,26] and this could be another reason why we did not find a significantly behavioral and only small central modulatory effects of ASA on pain processing structures.

## Conclusion

Taken together, our study replicates earlier findings of a possibly central effect of ASA on pain processing structures. As the model used in the present study allows to investigate the central processing of trigeminal input and it is well known that the trigeminal innervation plays a pivotal role in the pain transmission of headache disorders like migraine, our results support earlier indications that ASA might exert its therapeutic effect in migraine through a combination of peripheral and central mechanisms.

## Abbreviations

ASA: Acetylsalicylic acid; ACC: Anterior cingulate cortex; fMRI: Functional magnetic resonance imaging; SEM: Standard error of the mean; COX: Cyclooxygenase.

## Competing interest

The authors declare that they have no competing interests.

## Authors’ contribution

IK: design of the study, data acquisition, analysis and interpretation of data, drafting of the manuscript. AM: conception and design of the study, analysis of data, revising the manuscript critically for important intellectual content. Both authors read and approved the final manuscript.
